# Vulnerability of Coastal Communities from Storm Surge and Flood Disasters

**DOI:** 10.3390/ijerph13020239

**Published:** 2016-02-19

**Authors:** Jejal Reddy Bathi, Himangshu S. Das

**Affiliations:** Department of Civil and Environmental Engineering, Jackson State University, 1400 Lynch Street, Jackson, MS 39217, USA; himangshu.s.das@jsums.edu

**Keywords:** flood, vulnerability, climate change, storm surge, coastal communities

## Abstract

Disasters in the form of coastal storms and hurricanes can be very destructive. Preparing for anticipated effects of such disasters can help reduce the public health and economic burden. Identifying vulnerable population groups can help prioritize resources for the most needed communities. This paper presents a quantitative framework for vulnerability measurement that incorporates both socioeconomic and flood inundation vulnerability. The approach is demonstrated for three coastal communities in Mississippi with census tracts being the study unit. The vulnerability results are illustrated as thematic maps for easy usage by planners and emergency responders to assist in prioritizing their actions to vulnerable populations during storm surge and flood disasters.

## 1. Introduction

Coastal communities are commonly attributed with lower geographic elevations and are often associated with higher population densities than that of inland communities [1]. For example, in 2010, over 123 million people, or 39 percent of the American population, lived in coastal counties representing less than 10 percent of the United States (U.S.) land area (excluding Alaska). From 1970 to 2010, an average 39% population increase in coastal counties has been recorded. The average US coastal counties population density of 446 persons per square mile (in 2010) is expected to increase by 37 persons per square mile by 2020, whereas the entire US population density will only increase by 11 persons per square mile [2]. Unfortunately, most coastal counties are vulnerable to natural disasters, particularly from intense hurricanes. Over the past decade, the U.S. Gulf Coast has been severely affected by multiple hurricanes, including Ivan, Katrina, Rita, Ike and many others. Recently, Superstorm Sandy devastated the New York/New Jersey region. North and South Carolina faced significant economic loss, environmental degradation and public life disruption from hurricane Joaquin. These coastal storms, combined with rising populations, often challenge disaster managers and city planners in protecting public property and human life [2].

Public health, both physical and mental, is expected to be impacted not only during an active disaster but also after the disaster [3]. For example, hurricane Katrina and the Deepwater Horizon oil spill in the Gulf Coast of America have shown chronic mental health effects. However, the extent of the effect was related to the extent of distribution to where people live and work as well as their family structure and social engagement [4,5]. In the case of the oil spill disaster, mental health problems persisted even one year after the spill. Anxiety and depression were clinically significant, particularly for people who continued to sustain spill-related income loss [6,7]. The literature demonstrated that elderly, female, African Americans, less educated, and non-home owners (or the poor) are more vulnerable and are disproportionately affected by Katrina-induced health problems years after the storm [3,7]. It is undoubtedly true that there are a number of contributing factors that are likely to impact coastal communities. For example, in 2005, hurricane Katrina destroyed wetlands, leading to elimination of crucial buffer zones, leaving once protected infrastructure and neighborhoods exposed to more intense future storms. Among these populations, many residents are elderly, a group that is particularly vulnerable to coastal storms and flooding [8]. For example, nearly 85% of people killed during, and in the immediate aftermath of, Hurricane Katrina were aged 51 and older, and almost half were older than 75 years of age. In addition, climate change is expected to accelerate flood risks in the coming decades as the sea levels rise due to global warming, which will further intensify storm surges [1,9]. The extent of the impact from climate change may also depend on local conditions as well as the severity of climate change projections. For example, existing coastal neighborhoods may encounter increased and frequent localized flooding; drainage systems are likely to be overloaded more frequently and severely (as the dated drainage systems are not build for projected climate change), causing backups and street flooding; and people’s mobility may be hindered due to inundation of low-lying feeder roads in coastal areas [10]. 

Proper coastal management to protect populations and community resources from potential flood damage requires a systematic assessment of vulnerability. Measuring the vulnerability of an area or a targeted population has been studied for decades to help community planning and emergency management. Vulnerability, often expressed using a vulnerability index, has been conceptualized in many different ways. When resources become scarce, such as during massive disasters, vulnerability measures are very important for effective allocation of resources to mitigate hazards (e.g., land use practices and building construction practices), improve emergency preparedness practices (e.g., detection and warning systems), help emergency response (e.g., food and medical assistance) and for recovery preparedness practices (e.g., diversified investments and hazard insurance). A number of previous studies have demonstrated various approaches for coastal vulnerability assessment (e.g., [9,11,12,13,14,15,16,17]). These assessments are done at multiple spatial (global to local) and temporal (short term to long term) scales. Typically, a vulnerability matrix is developed with available data suitable to a specific problem [9]. Unfortunately, no single universal metric or measurement tool can be developed or applied to fit all criteria. Developing any such tool is challenging due to ever-present definitional ambiguity, along with the dynamic nature of the temporal and spatial scales of analyses [18]. 

Socioeconomic indicators in a community are most commonly used in measuring social vulnerability. While it is important to characterize population by broad categories of dominant socioeconomic indicators, it is also important to understand how each indicator combines with others to generate interactive vulnerabilities [19]. This is noted by Cutter, *et al*. (2009) [19] as, “*selecting a single variable (e.g., race, gender, or poverty) does not adequately capture communities described as African American female-headed households below the poverty level, because not all African Americans are in poverty; not all female-headed households are African American; and not all people in poverty are females or female-headed households*”. Previous research has shown that some of the commonly used socioeconomic indicators are strongly correlated. Thus, it is important to use either a composite measure of social vulnerability or a subset of these indicators to measure the vulnerability of a community [19,20]. 

The vulnerability of a community to a flood hazard is commonly measured using socioeconomic indicators or calculating physical flood extents, however, their combined impact is often ignored. Geographical Information System (GIS) based approaches have been used to understand the coastal flood vulnerability by overlaying the hydrodynamic models predicted flood areas over land surface elevations. However, this approach does not incorporate socioeconomic vulnerability [21,22,23,24]. There are few studies that consider combined socioeconomic and physical vulnerability [25,26,27]. The vulnerability assessment is often complex, requiring significant amounts of data, such as surface elevation surveys and development of detail hydrodynamic models, which are expensive.

In this paper, we proposed a simple approach that combines socioeconomic indicators as well as physical flood extents in measuring the combined vulnerability of an area. Our approach of vulnerability measurement is demonstrated for coastal counties of Mississippi as a case study. To date, no comprehensive studies have been carried out to explore the impact of flooding on infrastructure and public health for these three coastal counties in Mississippi where thousands of vulnerable populations are exposed to periodic and intense storm flooding. Preparing for the broad range of anticipated effects of coastal storms and floods may help reduce the public health burden. Thus, the focus of this paper is to identify critical populations vulnerable to disasters in order to help planners and communities better understand the baseline health status of neighborhoods. While the vulnerability of Mississippi’s coastal community to current flooding conditions are presented in this paper, future research needs to explore the vulnerability of public health from projected climate change (e.g., sea level rise) and anticipated future flood scenarios. 

## 2. Data and Methods

### 2.1. Study Area 

The study area represents three coastal counties (Jackson, Harrison and Hancock) in Mississippi. Jackson County encompasses approximately 736 square miles and borders Alabama (AL) in the east. Harrison County, in the middle, encompasses an area of approximately 976 square miles, while the Hancock County encompasses an area of approximately 485 square miles and borders Louisiana (LA). Harrison County, which encompasses the Biloxi urban area, is the most populous among the three counties. The major roads in the study area are Interstate Highway 10 and U.S. Highway 90, both of which serve primary east–west traffic, while state highways provide north–south access. Portions of Jackson County have elevation in excess of 100 feet (NGVD 29) while the coastal ridge in the City of Bay St. Louis is has an elevation of approximately 20 feet (NGVD 29), which drops to nearly sea level in some parts of Hancock County. Pascagoula River and Pearl River are two major river systems. The primary reason for choosing these three counties is due to their proximity to the coast, which is exposed to historical storms. For example, a storm in 1909 caused a surge height of about 8 to 12 feet along the Mississippi coast costing 150 lives, while the hurricane in 1915 had a surge height of about 11.8 feet in parts of Hancock County to about 9.0 feet in Harrison County costing 175 lives. Following the 1909 and 1915 events, a seawall with elevation four feet to eight feet was built to minimize wave and surge damage from hurricanes [28,29]. Among the several hurricanes and coastal floods noted over the past century, hurricane Katrina, which struck in August 2005, was the most powerful and deadliest of all. Hurricane Katrina struck low-lying coastal plains that are particularly vulnerable to storm surge flooding, damaging 130,000 homes and took over 200 lives in Mississippi; most of which were along the three coastal counties [30,31]. 

**Figure 1:**
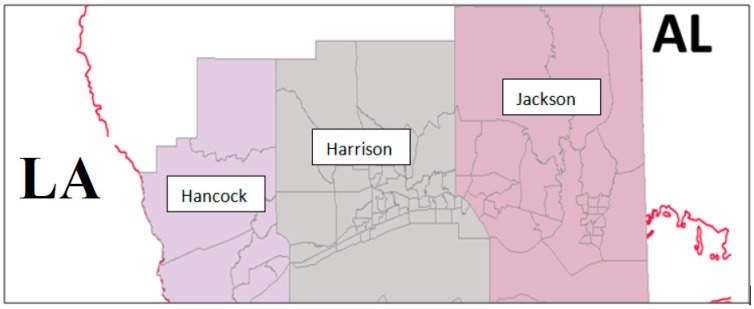
Study area showing census tracts in three MS coastal counties.

In total, there are 81 census tracts in these three counties, which are used in this study. Figure 1 illustrates the study area along with the census tracts. Table 1 shows selected socioeconomic variables of the different counties.

### 2.2. Methodology 

Vulnerability assessment is used to analyze the elements of exposure, susceptibility and resilience of any system to a hazard [1]. In this study, the vulnerable areas are identified as those census tracts that are occupied by demographic segments that are both socioeconomically disadvantaged and are most vulnerable to flood disasters. The vulnerable people in a community can be expressed in three closely linked ways: (1) social vulnerability (race, ethnicity, *etc.*); (2) economic vulnerability (income level); and (3) the climatological vulnerability (flood exposure). The first two vulnerabilities are combined as socioeconomic vulnerability and the third uses flood simulation data. This assessment is accomplished using GIS by overlaying socioeconomic data (e.g., poverty, minority populations and low education status) combined with hazard exposure (*i.e.*, flooding). The vulnerability of a community can be influenced by many factors, including socioeconomic factors such as demographics, income and education level, and the extent of hazard exposure. This indicates that not all people in hazard-exposed area are equally affected [20]. Our approach for calculating vulnerability of a census tract is to calculate average socioeconomic vulnerability normalized between 0 and 1, based on selected socioeconomic indicators of population in that tract. Flood exposure vulnerability is calculated based on the extent the tract is flooded during a flood disaster. The combined vulnerability of socioeconomic and flood vulnerabilities is calculated as an average of the two numbers. The socioeconomic indicators used and the methodology is discussed in the following sections. 

#### 2.2.1. Socioeconomic Vulnerability Indicators

Socioeconomic or social vulnerability arises from the potential for disaster to cause changes in people’s routine and lifestyle and their families based on their socioeconomic conditions [28]. As described by Federal Emergency Management Agency (FEMA) in Fundamentals of Emergency Management (AEMRC) [33] document, the socioeconomic vulnerability arises from various components, some of which can be predicted by demographical characteristics such as gender, age, education level, income, and ethnicity. GIS can be used to conduct disaggregated (e.g., census tract-level) spatial analyses to identify the demographic segments most likely to be vulnerable to disaster impacts. In addition, as the emergency managers, including health workers, have very limited access to direct measures of social vulnerability, geographic analyses of social vulnerability are conducted on Census data, preferably at the lowest possible level of aggregation (e.g., block-group or tract) [33]. 

People with the fewest psychological, social, economic, and political resources often disproportionately occupy the most hazardous geographical areas and the oldest, most poorly maintained buildings, which results in the greatest physical impacts such as casualties and property loss during a disaster [34]. The poor are less likely to have the income or assets needed to prepare for a possible disaster and for recovery efforts. While wealthy people may have higher monetary value of economic and material losses, the losses sustained by the poor are far more devastating in relative terms [19]. Similarly, people with higher education are expected to better prepare for a disaster and are less vulnerable to disaster impacts. Vulnerability of women increases not only due to their low income, in general, but also from their higher responsibilities because of their roles as mother and caregivers, which limits their ability to seek safety while caring for children and very old people who require assistance [19].

People that need physical help, especially those who are living in assisted living facilities (primarily people age 65 and more), and dependent children less than 18 years of age are the most vulnerable during a disaster cycle [34]. Lack of fluency in English language to understand disaster communication makes many immigrants, especially in rural communities, which attracts immigrates for agricultural and farming activity, more vulnerable to disasters [20,35]. Mobile homes, which are usually isolated with limited or no access to public transportation or highways, are more vulnerable to a hazard and such typical structures with no strong basement increases their vulnerability to severe weather and flooding [36]. Similarly, people living in multi-unit housing and high-rise apartments are more vulnerable due to their dense population limiting access and ability to evacuate [37,38]. No personal vehicle or access to public transportation restricts evacuation and hence is expected increase vulnerability to hazards. Overall, poor and minority populations (generally, non-white populations), and elderly nursing home residents, are more likely to lack transportation during disasters [39]. These populations often have a high prevalence of chronic health problems, which increases their vulnerability to other storm-related hazards [40]. 

Table 2 is presents a list of socioeconomic indicators adopted in the present study. Information related to socioeconomic indicators in the study area at census tract level is gathered from an ACS survey conducted in 2012. Each socioeconomic indicator was standardized (normalized) by dividing the indicator value for a tract by maximum value of the indicator in the study area. Standardization of indicators’ value is expected to create a comparative proportions among the indicators. An aggregate value of socioeconomic vulnerability of tracts was calculated as the average of standardized indicators values using the following Equation (1):

(1) $$Socioeconomic\;Vulnerability=\frac{\left[SSEI1+SSEI2+...+SSEI11\right]}{11}$$

where *SSEI*1–11 are standardized values for the 11 indicators listed in Table 2.

This yields aggregate vulnerability normalized between zero and one, which is also the same approach used in contemporary research [25]. 

#### 2.2.2. Flood (Climatological) Vulnerability Indicators

Geospatial data reported in FEMA’s National Flood Risk Report is used as an input data for computing flood hazard areas. Flood hazard areas on the (FIRM) are identified as a Special Flood Hazard Areas (SFHAs). SFHA are defined as an area that will be inundated by a flood event having a one-percent chance or a 100-year storm event, of being equaled or exceeded in any given year. The one-percent annual chance flood is also referred to as the base flood or 100-year flood. SFHAs are labeled as Zone A, Zone AO, Zone AH, Zones A1–A30, Zone AE, Zone A99, Zone AR, Zone AR/AE, Zone AR/AO, Zone AR/A1–A30, Zone AR/A, Zone V, Zone VE, and Zones V1–V30. Flood zones A, AO, AH, A1–A30, AE and A99 are high flood risk areas for 100-year flood. Zone AR represents areas with temporarily increased flood risk due to the building or restoration of a flood control system (such as a levee or a dam). Zones AR/AE, AR/AH, AR/AO, AR/A1–A30, and AR/A are dual flood zones that, because of flooding from other water sources that the flood protection system does not contain, will continue to be subject to flooding after the flood protection system is adequately restored. Zones V, VE and V1–V30 are areas along coasts subject to inundation by the one-percent-annual-chance flood event with additional hazards due to storm-induced velocity wave action. The areal extent of each flood zone (for FEMA flood, Special Flood Hazard Area (SFHA) by specific zones (e.g., A, AE, A1, A30, AH, AO, AR, A99, V, VE, and V1–V30) within each study census tract units was calculated through a spatial intersect process. Flood exposure area was calculated as the amount of land area within the 100-year flood zone as a fraction of the total land area in each census tract. These fractional values of the census tracts are assigned to represent the flooding vulnerability of the tracts. 

#### 2.2.3. Interactive Vulnerability Mapping

**Figure 2:**
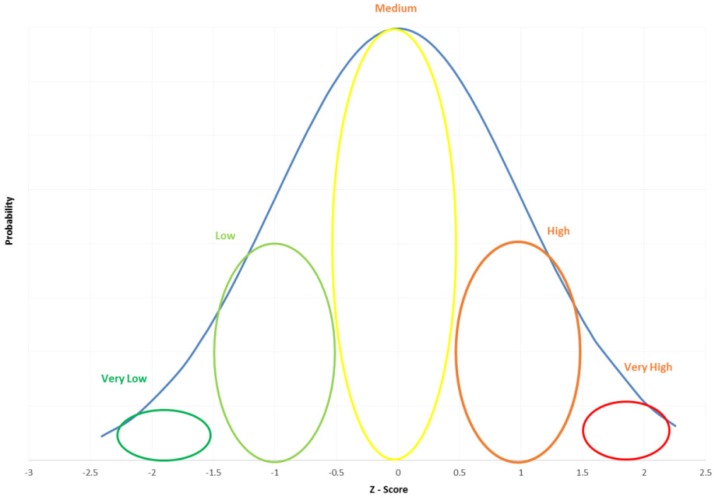
Use of Z-scores to determine vulnerability groups.

GIS mapping to generate thematic maps and the socioeconomic and climatological vulnerability scores is an effective way to visually identify areas of varying vulnerabilities. Similar to the approach summarized by the U.S. Army Corps of Engineers in the literature [41] for social vulnerability categories identification, normalized Z-scores of aggregate vulnerably values are used to identify tracts by vulnerability groups (Very Low, Low, Intermediate, High, and Very High). Final vulnerability score for each tract has been calculated as an average of the two vulnerability values (Socioeconomic and climatological). It is user specific to choose Z-score ranges to categories census tracts into individual vulnerability groups, which makes it easy to regroup the tracts into vulnerability groups depending on what is desired. For Z-scores distribution illustrated in Figure 2, tracts with Z-score of ≥1.5 and 0.5 < Z-score < 1.5 are assigned very high and high vulnerability, respectively, while all other tracts are intermediate to very low vulnerability. The Z-scores approach can help determine how close or far (standard deviations) a selected tract’s vulnerability is distributed compared to the mean vulnerability of tracts. For example, if the Z-score of a tract is –2.0, it indicates that the particular tract has two standard deviations lower vulnerability than the mean vulnerability of tracts in the study area. The same ranges of Z-scores shown in Figure 2 are used to assign tracts into vulnerability groups for the study area of coastal MS. 

## 3. Results 

**Figure 3:**
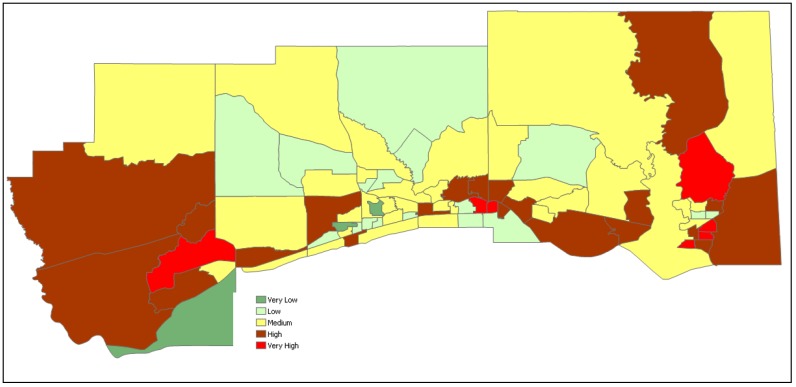
Relative vulnerability of tracts developed using normalized Z-score approach for coastal communities in MS.

As described in the methodology, an aggregate value of vulnerability for each census block has been calculated as an average of standardized indicators values normalized between zero and one. A final vulnerability score for each tract was calculated as the average of the two vulnerability values (socioeconomic and climatological). A thematic vulnerability map illustrating vulnerability of census tracts is shown in Figure 3. The interpretation of the thematic map is that, during a flood event, red or dark brown-colored census tracts are at higher risk than the tracts with light colors. In this particular case, there are seven census tracts found to be very highly vulnerable (Z-score ≥ 1.5), whereas 20 tracts are found to be highly vulnerable (0.5 ≤ Z-score < 1.5). The majority of tracts (32) are in the intermediate vulnerability group (−0.5 ≤ Z-score > 0.5), 16 tracts are in low vulnerability group (–1.5 ≤ Z-score < –0.5) and 16 tracts are in very low vulnerability group (Z-score < −1.5). 

Inland flooding caused by Pearl River, which drains to Gulf of Mexico, combined with higher population apparently cause the higher vulnerability of tracts in Jackson County relative to the other tracts along the Mississippi Coast. Although the most coastal tracts in Harrison County have considerable flooding, the overall combined vulnerability of these tracts is primarily low as the people in those tracts are mostly white, in the age group of 18 to 65, live in permanent structures and are educated. The majority of tracts in Hancock County are also highly vulnerable not only because of their lower elevation but also the population in the county is primarily under-educated and are low income people, who cannot afford to live in permanent housing or own a vehicle. 

The methodology presented in this paper for identifying vulnerable groups is very flexible. For example, the census tracts can be divided into four levels of vulnerability instead of the five presented in Figure 3. The vulnerability intervals can also be determined as equal quadruples of Z-scores or by other user specified criteria, which might produce varying vulnerability levels. For example, for the vulnerability assessment of the three coastal communities presented in this paper, if the vulnerability groupings were reassigned with Z-score ≥ 1.0 as very high vulnerability, 0.25 ≤ Z-score < 1.0 as high vulnerability, −0.25 ≤ Z-score < 0.25 as medium vulnerability, −1.0 ≤ Z-score > −0.25 as low vulnerability, and Z-score < –1.0 as very low vulnerability, the resulting tracks in each group would be 13, 20, 16, 19 and 13, respectively. 

**Figure 4:**
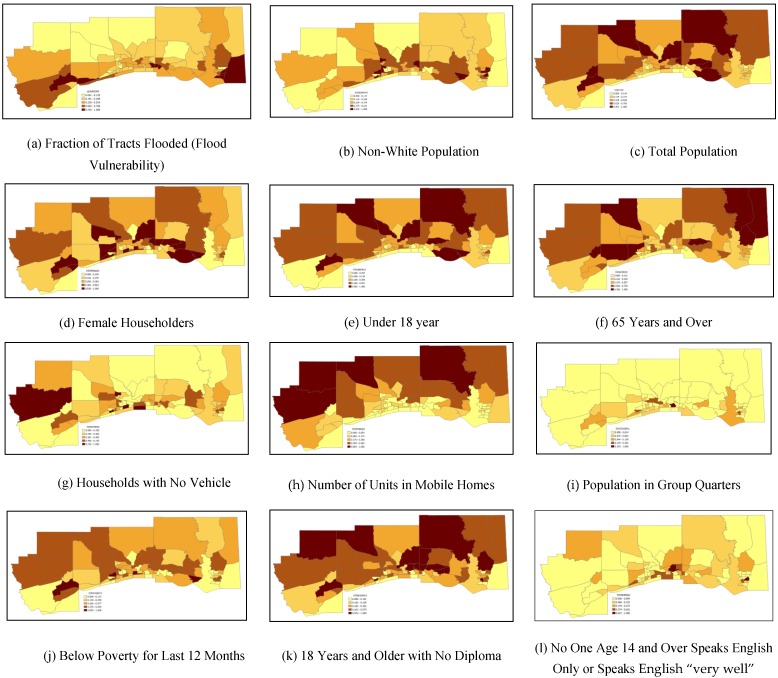
Relative vulnerability maps showing flood vulnerability and individual socioeconomic indicators vulnerability, with each indicator considered separately to measure vulnerability.

The vulnerability of a community exposed to a flood hazard determined based on a single indicator might be different than the actual vulnerability that is influenced by other factors. For example, the extent of disaster exposure may not be the only determining factor to assess the vulnerability of people in a community. Interaction of socioeconomic factors and the exposure to flood hazard are expected to impact the overall vulnerability. For example, vulnerability of census tracts taking only one indicator at time is illustrated in Figure 4. As a note, vulnerability of census tracts indicated in Figure 4 change from dark brown shaded census tracts being the highest vulnerability to pale yellow shaded tracts with lowest vulnerability. The 100-year flood exposure as fraction of census tracts flooded (flood vulnerability) is presented in Figure 4a. Based on flood exposure as the only vulnerability indicator, most of the census tracts in Harrison County are relatively highly vulnerable, which is different than the combined vulnerability of socioeconomic and flood vulnerability shown in Figure 3. Similarly, normalized non-white population distribution among census tracts is shown in Figure 4b, indicating the census tracts in Harrison County are found to have medium to high level of vulnerability. However, the combined vulnerability presented in Figure 3 indicated primarily medium to low vulnerability in Harrison County. Similarly, vulnerabilities of other socioeconomic indicators are presented in Figure 4c–l. How individual variables indicate vulnerability of a community is discussed Section 2.2.1.

The information presented in Figure 4 is not only useful for insight into the drivers of overall vulnerability of an area but is also useful to planners, emergency responders and others involved throughout a disaster cycle to target their actions if a specific indicator is of their interest. For example, if there is an epidemiological outbreak in which older (senior citizen) people are deemed to be impacted, then the medical emergency team can use the vulnerability map of indicator 65 years and above (Figure 4f) to prioritize their emergency actions. As another example, during a high wind event, the cluster of mobile home units (Figure 4h) will be of interest for decision makers and responders.

## 4. Conclusions

Post disaster, especially post Katrina, it was found that not all people are affected to same extent during and after a hazard [20]. For example, even a decade later, debate about the varied effects of Katrina and the failure of planning and response in certain demographical communities of the disaster area is still ongoing. The debate is on a claim that the communities that are primarily populated with minorities and low-income people were more heavily impacted from Katrina and thus these areas should have received priority in disaster response and mitigation efforts. This past experience shows that several socioeconomic factors, such as race and ethnicity, income, living conditions and education level, are some of the important indicators of vulnerability of people to a disaster. In addition, pre-existing health conditions are also expected to determine the extent of health related impacts from a hazard. Communities with pre-existing a health history will be more vulnerable than other communities. In that sense, the combined vulnerability as a result of interaction between socioeconomic, health and flood extent are expected to be different than the vulnerability arising due to any individual indicator. 

Historically, studies have demonstrated various approaches for coastal vulnerability assessment. These studies have often determined the vulnerability of a community based on a single socioeconomic indicator or in some cases a combination of socioeconomic indicators. For flood disasters, agencies most commonly assign the vulnerability of communities simply based on projected flooding of communities, ignoring socioeconomic conditions of the communities. There is limited information on community vulnerability assessment that combines socioeconomic and flood vulnerabilities. In this paper, we presented an assessment framework that combines socioeconomic vulnerability with flood vulnerability in determining the overall vulnerability of a community from flood disaster. This framework can be potentially incorporated into the development of more advanced decision support techniques. As a case study, the vulnerability assessment framework presented in the paper is demonstrated through calculation of vulnerability of census tracts in three Mississippi coastal counties and the results are presented as a thematic vulnerability map. Such thematic maps are useful for decision makers as well for the first responders who often make rapid decisions on needed action with limited access to data sources.

The approach used in this paper is based on a set of socioeconomic indicators that were used in contemporary research focusing on vulnerability from coastal floods. Often the socioeconomic indicators are expected to be interdependent with variation in one indicator’s value affecting the other indicator in the group. However, the framework presented in this paper is expected to minimize the bias that might arise from correlated indicators, as the framework includes calculating composite scores of socioeconomic indicators before combining with flood vulnerability score. In addition, the actual selection of indicators to apply for a given vulnerability assessment is expected to depend on many factors, most notably the purpose and scale of the vulnerability assessment and data availability. The socioeconomic indicators considered in this paper for assessing vulnerability for three coastal counties are primarily based on the fact that they are the most commonly used indicators in the literature for socioeconomic vulnerability assessment as well the data for these indicators are readily available from ACS. Users may select indicators that are relevant to their community. Each community has certain characteristics that make some indicators more suitable than others. For example, if a community has few or no mobile homes, then the people living in mobile homes may not be a required indicator in the community vulnerability assessment. Similarly, if the interest of assessment is to understand chemical or toxins exposure vulnerability during a flood disaster, then one need to consider presence of toxic material storage facilities, solid waste storage units, wastewater treatment units and hazardous chemical processing industries and their material storage locations in assessment. Some other socioeconomic and health indicators of interest may include accessibility of roads, availability of healthcare facilities, number and location of assisted living facilities, college student dormitories and workers dormitories, emergency shelters, *etc.*

The socioeconomic vulnerability calculation approach presented in this paper assumes all indicators are weighted equally in the vulnerability assessment. However, if the user has specific information that confirms that, for their community, some indicators have higher vulnerability than other, then the user can assign appropriate multiplication factors to adjust for higher vulnerability of some indicators over others.

As the framework presented in this paper is based on indicators, the accuracy of combined vulnerability depends on the accuracy of the indicators’ data. It is also important that potential discrepancies in the results be noted. However, as the approach allows for relative vulnerability comparison among the study units, uncertainties are assumed to be equally weighed. In this way, uncertainty is not removed, but is integrated into the assessment. Overall, the successful assessment of vulnerability is predicated on the generation of a comprehensive set of vulnerability metrics that fully and accurately describe the exposure [41]. 

As a continuation of the current research presented in this paper, we plan to explore quantification of vulnerability in public health sector from projected climate change scenarios. Climate change is an area of intense research and has gradually evolved into a multidisciplinary field. In the public health sector, impact from climate change can be influenced by many factors, including the severity as well as other characteristics of storms, such as the exact timing and location of impact and the unique geographic and topographic characteristics of the affected area. The next phase of our study is to augment the systematic approach that we developed here with future climate change scenarios of coastal communities. The focus of our planned research will be to identify public health indicators (such as existing diseases, age, *etc.*) and health infrastructure in coastal counties to assess the public health vulnerability from projected climate change scenarios. 

## Figures and Tables

**Table 1:** Key demographic information of the study area (Source: American Community Survey (ACS)) [32].

Statistic	Jackson County	Harrison County	Hancock County
Total Population	139,430	188,110	44,044
Non-White Population	37,259	54,794	5244
Female Population	70,740	94,766	22,401
Population < 18 years	35,338	46,078	10,423
Population > 65 years with Disability	7859	9476	2373
Population Below Poverty	21,238	33,162	8572
Average Household Income	47,266	42,060	42,028

**Table 2:** Socioeconomic vulnerability indicators adopted in this study.

1	Total Population
2	Non-White Population
3	Number of Female Households
4	Population Under 18 Years
5	Population 65 Years and Older or Disabled
6	Households with No Vehicle
7	Housing Units in Mobile Homes
8	People in Group Quarters
9	People Below Poverty in Past 12 Months
10	Population 18 Years and Over with No Diploma
11	No One Age 14 and Over Speaks English Only or Speaks English “very well”;
